# HLA Class II Alleles Susceptibility Markers of Type 1 Diabetes Fail to Specify Phenotypes of Ketosis-Prone Diabetes in Adult Tunisian Patients

**DOI:** 10.1155/2011/964160

**Published:** 2011-03-02

**Authors:** Lilia Laadhar, Fatma Harzallah, Mondher Zitouni, Maryam Kallel-Sellami, Moncef Fekih, Naziha Kaabachi, Hádia Slimane, Sondès Makni

**Affiliations:** ^1^Immunology Department, La Rabta Hospital and Al Manar University Tunis, 1007 Tunis, Tunisia; ^2^Endocrinology Department, La Rabta Hospital and Al Manar University Tunis, 1007 Tunis, Tunisia; ^3^Biochemistry Department, La Rabta Hospital and Al Manar University Tunis, 1007 Tunis, Tunisia

## Abstract

We aimed to characterize the different subgroups of ketosis-prone diabetes (KPD) in a sample of Tunisian patients using the A*β* scheme based on the presence or absence of *β*-cell autoantibodies (A+ or A−) and *β*-cell functional reserve (*β*+ or *β*−) and we investigated whether HLA class II alleles could contribute to distinct KPD phenotypes. We enrolled 43 adult patients with a first episode of ketosis. For all patients we evaluated clinical parameters, *β*-cell autoimmunity, *β*-cell function and HLA class II alleles. Frequency distribution of the 4 subgroups was 23.3% A+*β*−, 23.3% A−*β*−, 11.6% A+*β*+ and 41.9% A−*β*+. Patients from the group A+*β*− were significantly younger than those from the group A−*β*− (*P* = .002). HLA susceptibility markers were significantly more frequent in patients with autoantibodies (*P* = .003). These patients also had resistance alleles but they were more frequent in A+*β*+ than A+*β*− patients (*P* = .04). Insulin requirement was not associated to the presence or the absence of HLA susceptibility markers. HLA class II alleles associated with susceptibility to autoimmune diabetes have not allowed us to further define Tunisian KPD groups. However, high prevalence of HLA resistance alleles in our patients may reflect a particular genetic background of Tunisian KPD population.

## 1. Introduction

According to the American Diabetes Association [1], there are 2 categories of type 1 diabetes, type 1A or immune-mediated diabetes and type 1B or idiopathic diabetes. The first category is defined by the presence of HLA risk markers and at least one of these autoantibodies: ICAs (islet cell antibodies), anti-GAD (glutamate decarboxylase), anti-IA2 (islet antigen 2), and anti-insulin antibodies. Patients from the second group have neither *β*-cell autoimmunity markers nor HLA predisposing alleles. 

Diabetic ketoacidosis has been traditionally considered as a complication of type 1 diabetes. However, it could occur in patients with apparently heterogeneous forms of diabetes [2]. The etiological bases of ketosis-prone diabetes (KPD) are unknown. Several methods of classification of these forms were described [3–6] and the most accurate in predicting long-term *β*-cell function and clinical outcome was the A*β* scheme [7–9]. It is a classification based on presence or absence of auto-immune markers (A+ or A−) and of *β*-cell functional reserve (*β*+ or *β*−). According to this classification we could distinct 4 groups (A+*β*+, A−*β*+, A+*β*−, and A−*β*−). This scheme was widely used in American patients with multiethnic origin, and we were the first to use this scheme to classify North-African patients with KPD [10].

Although this classification had high sensitivity and specificity to predict long-life insulin requirement for these patients, evolution could be different in the same group. Genetic factors could contribute to these phenotypic differences. In fact, it is widely believed that some HLA class II alleles are associated with susceptibility or resistance to autoimmune diabetes in several populations [11, 12]. In Tunisia, it has been shown that HLA DRB1∗03, DRB1∗04, DQB1∗0201, and DQB1∗0302 are risk alleles for type 1 diabetes, while HLA DRB1∗11, DRB1∗15, DQB1∗06, and DQB1∗0301 are protective alleles [13, 14].

We aimed in this study to characterize the different subgroups of KPD patients in a sample of Tunisian patients using the A*β* scheme, and we investigated whether HLA class II alleles (DR and DQ) associated with susceptibility or resistance to autoimmune diabetes could contribute to distinct KPD phenotypes.

## 2. Patients and Methods

The protocol was approved by the ethical committee of La Rabta Hospital (Tunis, Tunisia) and informed consent was obtained from all participants. 

During two years, we recruited all adult patients (>30 years) admitted to the Endocrinology Department of La Rabta Hospital with a first episode of ketosis (without any history of secondary diabetes, steroid treatment, pregnancy, or infectious disease). Ketosis onset was defined as the presence of hyperglycemia (>2 g/l), ketonuria (HCO3*‒*< 15 mEq/l, pH < 7.30 on arterial blood sample), and immediate need for insulin treatment without previously known diabetes. 

All patients underwent a detailed assessment of medical history, physical signs, and serum and urine biochemistry. The weight status was classified on the basis of body mass index (BMI). After the resolution of the ketosis episode, patients were monitored for at least 6 months (maximum 2 years). 

ICAs were detected by indirect immunofluorescence using monkey pancreas section (the Binding Site, UK). Anti-GAD and anti-IA2 were detected by radio-immunoprecipitation (Immunotech, France). C-peptide was measured in serum within one week after ketosis resolution by radio-immunoprecipitation (Immunotech, France) at initial presentation and 6 minutes after glucagon stimulation. Insulin secretion was considered preserved when C-peptide was higher than 1 ng/ml at baseline or 1.5 ng/ml after stimulation.

HLA-DRB1 and DQB1 alleles were typed by PCR sequence-specific primer (micro-SSP 2L, One Lambda, USA) according to laboratory procedures.

Statistical analysis was performed using SPSS 11.5. For means comparison, we used Snedecor *F* test with Bonferroni correction where appropriate. Fisher's exact test was used to compare allele frequencies. For all statistical tests, *P* ≤ .05 was considered significant.

## 3. Results

We enrolled in this prospective study 43 patients (25 men and 18 women). The mean age was 47 ± 12.1 years. 

Fifteen out of these 43 patients (34.8%) had at least one positive autoantibody, 32 (74.4%) had HLA risk markers of type 1 diabetes, and 23 (54.4%) had a correct *β*-cell function.

According to the A*β* scheme, frequency distribution of the 4 subgroups was 23.3% A+*β*−, 23.3% A−*β*−, 11.6% A+*β*+, and 41.8% A−*β*+. Their demographic and clinical characteristics are shown in Table 1. Patients from the group A+*β*− were significantly younger than those from the group A−*β*− (*P* =  .002). There were no significant differences in sex, familial history of diabetes, or BMI distribution across the 4 subgroups.

After six months, all patients of *β*− groups remained on insulin treatment. Insulin was successfully discontinued in 26% of patients of *β*+ groups and all of them remained on oral agents until the end of the study. 

**Figure 1:**
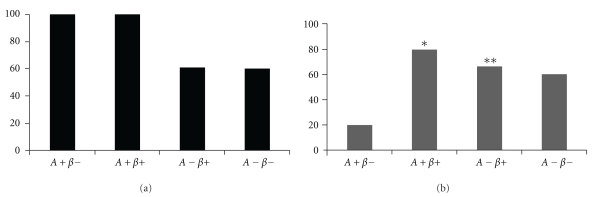
Susceptibility (a) and resistance (b) HLA class II markers in the KPD subgroups. (a) Frequencies of type 1 diabetes susceptibility alleles were 100%, 100%, 61.1%, and 60% in the A+*β*−, A+*β*+, A−*β*+, and A−*β*− groups, respectively. (b) Frequencies of type 1 diabetes resistance alleles were 20%, 80%, 66.6%, and 60% in the A+*β*−, A+*β*+, A−*β*+, and A−*β*− groups, respectively. *  *P* = .04 for A+*β*− compared with A+*β*+ groups. **P = .04 for A+*β*− compared with A−*β*− groups.

The distribution of HLA susceptibility and resistance markers to type 1 diabetes in the 4 subgroups is shown in Figure 1. There is no significant difference among the HLA susceptibility markers between these four subgroups. However, HLA susceptibility markers were significantly more frequent in patients with autoantibodies (*P* = .003). Patients from A+ subgroups also had resistance alleles but they were more frequent in A+*β*+ than A+*β*− patients (80% versus 20%, *P* = .04).

Compared with the two *β*− groups, the two groups with preserved *β*-cell functional reserve had a higher frequency of resistance alleles (68.3% versus 40%, *P* =  ns) and the same frequency of susceptibility alleles (60%).

If we consider every marker alone (Table 2), we found that the susceptibility allele DQB1∗0201 was significantly more frequent in patients from *β*− subgroups (*P* = .03). Within the *β*− groups, its prevalence was significantly higher in A+*β*− than A−*β*− patients (*P* = .02). DQB1∗0201 was also found significantly more common in patients from A+ subgroups (*P* = .001). 

Concerning resistance alleles, DQB1∗0301 was significantly more frequent in patients from *β*+ subgroups (32.6% versus 12.5%, *P* = .02). 

In order to better identify the type of diabetes in our patients we classify them using the A*β* scheme associated with HLA markers (Table 3). Patients with HLA susceptibility markers were considered as HLA+ and those without these markers were considered as HLA−.

After six months of followup, insulin was successfully stopped in only one patient of A+ groups who was A+*β*+HLA+; this patient had also resistance alleles. All A−*β*− patients remained on insulin treatment. Five out of 11 patients from A−*β*+HLA+ patients were under oral agents and 6/7 of A−*β*+HLA− patients remained on insulin treatment.

## 4. Discussion

In clinical practice, ketosis-onset diabetes in adults is a rare situation. In fact during two years only 43 patients were enrolled in this study. Correct classification of diabetes type at the time of diagnosis in such patients is often difficult but is clearly important in decisions regarding long-term management. In these patients, recognition of metabolic, immunological, and genetic markers of type 1 diabetes will allow patients to remain on insulin therapy because diet and oral pharmacological therapy are likely to fail [15].

In this prospective study we used the A*β* scheme and HLA susceptibility markers to classify our patients presenting with first episode of ketosis. 

Proportion of patients who were A+*β*− was 23.2%. All of them had HLA susceptibility markers and remained on insulin treatment after 6 months of followup. These patients are likely identical with the well-defined form of autoimmune type 1 diabetes. They were the youngest patients. Despite being more frequent in childhood patients, ketosis-onset diabetes in adults has already been reported in other studies with different ethnic origins [5, 16]. We have already defined this type of patients in a previous study [10]. 

Recently, using data from the Tunisian National Nutrition Survey, Bouguerra et al. reported that the prevalence of adult-onset diabetes is increasing [17]. Our study suggests that autoimmune type 1 diabetes may contribute to the increasing incidence of diabetes in the Tunisian adult population.

Only five patients were A+*β*+ (11.6%), and there is a real confusion in the literature concerning the classification of these patients. According to several European studies, these patients could be considered as antibody-positive type 2 diabetes or latent autoimmune diabetes of adults (LADA) [18–20]. Probably the greatest area of confusion involves the distinction of LADA from type 1 diabetes initially diagnosed in individuals over the age of 30 years. This form is characterized by the fact that patients can be initially managed with diet and oral hypoglycemic agents before becoming insulin dependent. 

It is interesting to note that all A+*β*+ patients had HLA susceptibility markers which make the diagnosis of autoimmune diabetes more plausible. Nevertheless, there is some support for the view that LADA shares susceptibility genes with type 1 diabetes [18, 21–23]. On the other hand, 80% of A+*β*+ patients also had resistance alleles. Coexistence of susceptibility and resistance alleles was not assessed in LADA patients in the literature. We cannot explain the exact role this combination plays in the pathogenesis of diabetes.

In the A+*β*− and A+*β*+ groups, we noticed that HLA susceptibility markers were not useful for the classification of patients, since they were strongly correlated to antibody production which has already been reported [24, 25]. However, HLA resistance markers were significantly higher in A+*β*+ group (*P* =  .04). This fact should be investigated further to see if these genetic factors could contribute to the delay of *β*-cell destruction by an immunologic mechanism. 

We find that the largest group was A−*β*+ (41.9 %) which was already reported in other studies [5, 26]. A−*β*+ patients appear clinically heterogeneous with a wide range of age and BMI (Table 1). In this group ketosis could be explained by a functional and partially reversible *β*-cell deficiency in type 2 diabetes patients secondary to glucotoxicity [27] or lipotoxicity [28]. KPD in type 2 diabetes has been first reported in black populations [29, 30] then in other ethnic groups [5]. 

After six months of followup, 13/18 (72.2%) of A−*β*+ patients remained on insulin treatment; this high prevalence has already been reported by Nalini et al. [31] after 12 months of followup. 

We noted a high frequency of both autoimmune diabetes susceptibility and resistance alleles in A−*β*+ patients. This was also reported by Nalini et al. [26] in a heterogeneous population with multiethnic origin (African American, Hispanic, Caucasian, and Asian KPD A−*β*+ patients). Among these patients, African American had the highest prevalence of autoimmune diabetes susceptibility and resistance alleles. Although, our population is white African and considered as Caucasoid, these similarities may be explained by shared African ancestry.

HLA markers seem to be not useful to distinguish patients in this heterogeneous group since there was no association between these markers and insulin requirement at six months (6/7 of A−*β*+HLA− patients remained on insulin treatment, and 5/11 patients from A−*β*+HLA+ patients were under oral agents). 

Other genetic markers were associated with propensity to KPD [32]. Nevertheless such markers are not suitable for routine diagnosis. 

Among our patients 23.2% were A−*β*−. *β*-cell failure in these patients could have different mechanisms including autoimmune and non-autoimmune ones. Autoimmune *β*-cell failure cannot be totally excluded in A−*β*− patients since circulating type 1 diabetes autoantibody levels decline over time to undetectable levels [33]. This is may be the case for six of our patients who were A−*β*−HLA+.

The four A−*β*−HLA− patients could have either long evolution type 2 diabetes or type 1B diabetes. This latter was first described in patients of African American origin [29] then in other ethnic groups (Asian, Native and Hispanic Americans, or European populations). No study has yet measured the rate of type 1B diabetes in the Tunisian population.

Irrespective to HLA alleles, all of the A−*β*− patients remained on insulin treatment after six months of followup suggesting that these markers are not suitable to better characterize these patients.

## 5. Conclusion

Atypical forms of diabetes have sparked a vigorous debate into the need for reliable markers for classification. Despite the restricted number of our patients, we can conclude that the A*β* scheme seems to be the strongest indicator of future metabolic control. HLA class II alleles associated with susceptibility to autoimmune diabetes have not allowed us to further define Tunisian KPD groups. However, high prevalence of HLA resistance alleles in our patients may reflect a particular genetic background of Tunisian KPD population. Further studies on a larger cohort are needed to search the ideal marker to predict the evolution of KPD patients. Special interest should be given to the implication of HLA resistance alleles in the physiopathology of this heterogeneous form of diabetes in association with other genetic markers.

## Figures and Tables

**Table 1:** Demographic and clinical characteristics of the 4 KPD subgroups.

	A+*β*−	A+*β*+	A−*β*+	A−*β*−	*P*
*N* (%)	10 (23.3)	5 (11.6)	18 (41.8)	10 (23.3)	—
Age (years)	36.3 ± 4.9	47 ± 3.8	46.6 ± 10.9	54 ± 1.4	.005
Men-to-women ratio	8/2	3/2	11/7	6/4	ns
Family history of type 1 diabetes (%)	6 (60)	5 (60)	9 (50)	6 (60)	ns
BMI (Kg/m^2^)	24.2 ± 5.2	29.8 ± 4.1	25.5 ± 5.2	25 ± 4	ns
C-peptide at baseline (ng/ml)	0.39 ± 0.29	1.42 ± 0.45	1.68 ± 0.76	0.48 ± 0.27	<.0001
C-peptide after stimulation(ng/ml)	0.55 ± 0.31	2.07 ± 1.03	2.10 ± 1.04	0.71 ± 0.35	<.0001
Insulin requirement at 6 months	10 (100)	4 (80)	13 (72.1)	10 (100)	ns

ns: non significant and BMI: body mass index.

**Table 2:** HLA class II allele frequencies in KPD subgroups.

HLA allele	A+*β*− 2*n* = 20 *N* (%)	A−*β*− 2*n* = 20 *N* (%)	A+*β*+ 2*n* = 10 *N* (%)	A−*β*+ 2*n* = 36 *N* (%)
Susceptibility				
DRB1∗03	6 (30)	3 (15)	2 (20)	6 (16.6)
DRB1∗04	4 (20)	3 (15)	3 (30)	5 (13.8)
DQB1∗0201	12 (60)	4 (20)	3 (30)	6 (16.6)
DQB1∗0302	4 (20)	2 (10)	3 (30)	8 (22.2)

Resistance				
DRB1∗11	1 (5)	3 (15)	1 (10)	7 (19.4)
DRB1∗15	2 (10)	2 (10)	0	2 (5.5)
DQB1∗06	1 (5)	3 (15)	0	6 (16.6)
DQB1∗0301	1 (5)	4 (20)	4 (40)	11 (30.5)

**Table 3:** KPD subgroups according to HLA susceptibility markers.

Autoantibodies A	*β* function	Susceptibility HLA	Nb	%
+	+	+	5	11.6
+	+	−	0	0
+	−	+	10	23.3
+	−	−	0	0
−	+	+	11	25.6
−	+	−	7	16.3
−	−	+	6	13.9
−	−	−	4	9.3
